# Parsing the synonymous mutations in the maize genome: isoaccepting mutations are more advantageous in regions with codon co-occurrence bias

**DOI:** 10.1186/s12870-019-2050-1

**Published:** 2019-10-14

**Authors:** Duan Chu, Lai Wei

**Affiliations:** College of Life Sciences, Beijing Normal University, No. 19 Xinjiekouwai Street, Haidian District, Beijing, China

**Keywords:** Synonymous mutations, Isoaccepting mutations, Codon co-occurrence bias, Maize (*Zea mays*), Natural selection

## Abstract

**Background:**

Synonymous mutations do not change amino acids but do sometimes change the tRNAs (anticodons) that decode a particular codon. An isoaccepting codon is a synonymous codon that shares the same tRNA. If a mutated codon could base pair with the same anticodon as the original, the mutation is termed an isoaccepting mutation. An interesting but less-studied type of codon bias is codon co-occurrence bias. There is a trend to cluster the isoaccepting codons in the genome. The proposed advantage of codon co-occurrence bias is that the tRNA released from the ribosome E site could be quickly recharged and subsequently decode the following isoaccepting codons. This advantage would enhance translation efficiency. In plant species, whether there are signals of positive selection on isoaccepting mutations in the codon co-occurred regions has not been studied.

**Results:**

We termed polymorphic mutations in coding regions using publicly available RNA-seq data in maize (*Zea mays*). Next, we classified all synonymous mutations into three categories according to the context, i.e., the relationship between the focal codon and the previous codon, as follows: isoaccepting, nonisoaccepting and nonsynonymous. We observed higher fractions of isoaccepting mutations in the isoaccepting context. If we looked at the minor allele frequency (MAF) spectrum, the isoaccepting mutations have a higher MAF in the isoaccepting context than that in other regions, and accordingly, the nonisoaccepting mutations have a higher MAF in the nonisoaccepting context.

**Conclusion:**

Our results indicate that in regions with codon co-occurrence bias, natural selection maintains this pattern by suppressing the nonisoaccepting mutations. However, if the consecutive codons are nonisoaccepting, mutations tend to switch these codons to become isoaccepting. Our study demonstrates that the codon co-occurrence bias in the maize genome is selectively maintained by natural selection and that the advantage of this trend could potentially be the rapid recharging and reuse of tRNAs to increase translation efficiency.

## Background

**Fig. 1:**
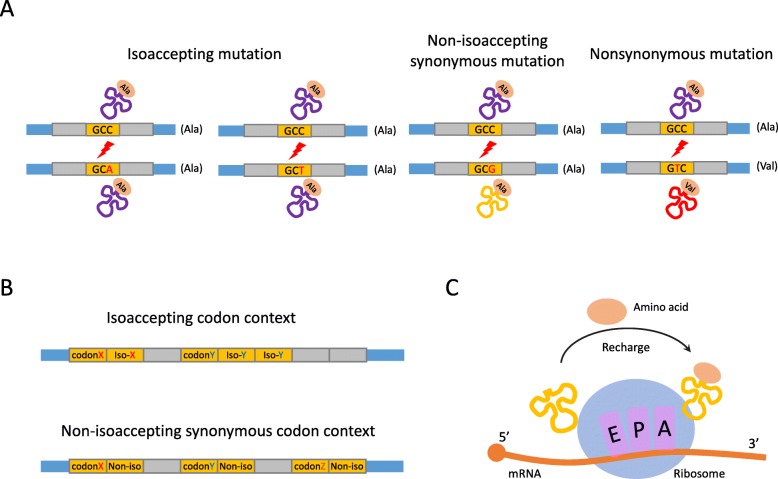
A summary of the methods and materials used in this study. **a** Classification of mutations in CDS according to their functional consequences. Synonymous mutations do not change amino acids while nonsynonymous mutations alter amino acids. Synonymous mutations are further divided into isoaccepting and nonisoaccepting mutations. Codons with an isoaccepting mutation could still base pair with the original anticodons (tRNAs). Nonisoaccepting mutations lead to base pairing with a different anticodon (tRNA). **b** Definition of the terminologies describing codon co-occurrence in this study. Isoaccepting codon stretches are the regions with consecutive isoaccepting codons. The same goes for nonisoaccepting stretches. **c** The proposed explanation for the advantage of codon co-occurrence bias. The tRNA released from the ribosome E site is rapidly recharged by the aminoacyl-tRNA synthetase. The recharged tRNA could be immediately used by the following isoaccepting codons

Synonymous mutations are mutations in CDS that do not change amino acid (AA) sequences. However, since different tRNAs (or anticodons) might carry the same AA, the unchanged AA does not necessarily ensure an unchanged tRNA (anticodon). This confounding situation is resolved by the terminology “isoaccepting codons”. An isoaccepting codon is a synonymous codon that shares the same tRNA (and obviously loads the same AA) with a different codon [1, 2] (Table 1). Similarly, the term “nonisoaccepting synonymous codons” represents codons encoding the same AA but that never share the same tRNA (anticodon). If a mutated codon could base pair with the same anticodon as the original, this mutation is defined as an isoaccepting mutation (Fig. 1a). In contrast, nonisoaccepting synonymous mutations do not alter the encoding AA but lead to a different decoding tRNA (Fig. 1a). Of note, a nonsynonymous mutation is also a nonisoaccepting mutation by definition because it alters the decoding tRNA (Fig. 1a). However, to avoid potential ambiguity, in this study, the nonisoaccepting mutation only refers to the synonymous mutation that changes the tRNA.

**Table 1:** List of the isoaccepting codon(s) for a given codon

Codon	Isoaccepting codon(s) (apart from the codon itself)	
	All possible	*Zea mays*
AAA	AAG	AAG
AAC	AAT	AAT
AAG	AAA	AAA
AAT	AAC	AAC
ACA	ACC/ACT/ACG	ACC/ACT/ACG
ACC	ACA/ACT	ACA/ACT
ACG	ACA	ACA
ACT	ACA/ACC	ACA/ACC
AGA	AGG	AGG
AGC	AGT	AGT
AGG	AGA	AGA
AGT	AGC	AGC
ATA	ATT/ATC	ATT/ATC
ATC	ATA/ATT	ATA/ATT
ATT	ATA/ATC	ATA/ATC
CAA	CAG	CAG
CAC	CAT	CAT
CAG	CAA	CAA
CAT	CAC	CAC
CCA	CCC/CCT/CCG	CCC/CCT/CCG
CCC	CCA/CCT	CCA/CCT
CCG	CCA	CCA
CCT	CCC/CCA	CCC/CCA
CGA	CGC/CGT/CGG	CGC/CGT/CGG
CGC	CGA/CGT	CGA/CGT
CGG	CGA	CGA
CGT	CGC/CGA	CGC/CGA
CTA	CTT/CTC/CTG	CTT/CTC/CTG
CTC	CTA/CTT	CTA/CTT
CTG	CTA	CTA
CTT	CTA/CTC	CTA/CTC
GAA	GAG	GAG
GAC	GAT	GAT
GAG	GAA	GAA
GAT	GAC	GAC
GCA	GCT/GCC/GCG	GCT/GCC/GCG
GCC	GCT/GCA	GCT/GCA
GCG	GCA	GCA
GCT	GCA/GCC	GCA/GCC
GGA	GGC/GGT/GGG	GGG
GGC	GGA/GGT	GGT
GGG	GGA	GGA
GGT	GGC/GGA	GGC
GTA	GTC/GTT/GTG	GTC/GTT/GTG
GTC	GTT/GTA	GTT/GTA
GTG	GTA	GTA
GTT	GTC/GTA	GTC/GTA
TAC	TAT	TAT
TAT	TAC	TAC
TCA	TCC/TCT/TCG	TCC/TCT/TCG
TCC	TCA/TCT	TCA/TCT
TCG	TCA	TCA
TCT	TCA/TCC	TCA/TCC
TGC	TGT	TGT
TGT	TGC	TGC
TTA	TTG	TTG
TTC	TTT	TTT
TTG	TTA	TTA
TTT	TTC	TTC

Codon bias usually refers to the unequal usage of synonymous codons by the genome, which is specifically termed codon usage bias (CUB) [3]. CUB is prevalent in all organisms, and selection acting on synonymous mutations has been widely revealed [4, 5]. The biological significance of CUB might be its impact on the mRNA translation elongation process [6–9]. Although the major determinant of the translation elongation rate is still under debate [10–17], it is commonly accepted that optimized/favored codons are generally translated faster than those that are unfavorable. This advantage of fast translating codons is especially useful during rapid cell growth/division [18].

Another less-studied type of codon bias is the codon co-occurrence bias. It was found that the order of codon occurrence inside a gene is biased. In yeast, the synonymous codons recognized by the same type of tRNA tend to be clustered in the coding region [1]. These stretches of codons could be either identical codons or isoaccepting codons (Fig. 1b). The advantage of this co-occurrence bias is that the tRNAs could be quickly recycled. These tRNAs are recharged by aminoacyl-tRNA synthetases near the translating ribosomes and are rapidly reused for the decoding of the following isoaccepting codons [1] (Fig. 1c). The accessibility of tRNAs is an important factor that affects translation elongation speed. The rapid recharging and reuse of tRNAs has positive effects on the local translation efficiency.

This nonrandom distribution of codon orders was verified in bacteria and yeast. However, in the plant kingdom, the most widely studied type of codon bias is the codon usage bias (CUB) [19–22]. Systematic and multispecies studies on codon co-occurrence bias are still lacking. Importantly, the proposed advantage of “co-occurred isoaccepting codons” is the rapid recharging and reuse of tRNA. If this assumption is true, we should observe more isoaccepting mutations than nonisoaccepting mutations in the codon co-occurring regions. The nonisoaccepting mutations in these regions would abolish the rapid recycling of tRNAs because the codons following the mutation are no longer isoaccepting.

We tested our hypothesis in the maize (*Zea mays*) genome. We extracted the polymorphic mutations in coding regions using publicly available RNA-seq data in maize. We then classified all synonymous mutations into three categories according to the environment, i.e., the relationship between the focal codon and the previous codon, as follows: isoaccepting, nonisoaccepting and nonsynonymous. We observed higher fractions of isoaccepting mutations in the isoaccepting context. If we looked at the minor allele frequency (MAF) spectrum, isoaccepting mutations have a higher MAF in the isoaccepting context than other regions. Accordingly, the nonisoaccepting mutations have a higher MAF in the nonisoaccepting context.

Our results demonstrate that in the regions containing co-occurring isoaccepting codons, natural selection maintains this co-occurrence pattern by suppressing nonisoaccepting mutations in these regions. However, if the consecutive codons themselves are nonisoaccepting (but synonymous), the mutations in these regions also tend to be nonisoaccepting (but synonymous). This mutation bias seems to switch (or fix) these nonisoaccepting codons to become isoaccepting.

At the genome-wide level, we have systematically characterized the codon co-occurrence bias in maize. The codon co-occurrence bias is selectively favored and maintained by natural selection. The advantage of this co-occurrence bias could be to promote the rapid recharging and reuse of tRNAs to increase translation efficiency. We propose that the biological significance of different types of codon bias (codon usage bias and codon co-occurrence bias) might result in fine-tuning of the translation elongation process. Intriguingly, given that codon co-occurrence bias might contribute to mRNA translation and that high translation efficiency is advantageous during rapid cell growth [18], we raise the question of whether this pattern is widespread in plant species.

Our work deepened the understanding of codon co-occurrence bias in plants from the perspective of evolutionary biology and might provide novel perspectives to help solve the riddles related to angiosperm evolution.

## Results

### Calling the polymorphic mutations in the CDS of maize

The polymorphic mutations in CDS were called by using publicly available RNA-seq data in maize root (Methods). We mapped the RNA-seq reads to the reference CDS sequence and called variants. Only those variation sites with a level between 0.02 and 0.98 were regarded as candidate polymorphic sites (Methods). We obtained 24,323 polymorphic mutation sites in CDS of maize (Additional file 1: Figure S1). Of note, these sites did not include those mutations taking place in the same codon or consecutive codons (Methods). The mutations in CDS might have different functional consequences, such as not changing amino acids (AAs), changing AAs, or inducing/damaging a stop codon, such that we need to classify these polymorphic mutations in CDS into different categories. Even if a synonymous mutation does not change the AA, it might possibly change the tRNA that pairs with the codon (an example of alanine codons is given in Additional file 1: Figure S2).

### Defining the mutation types

**Fig. 2:**
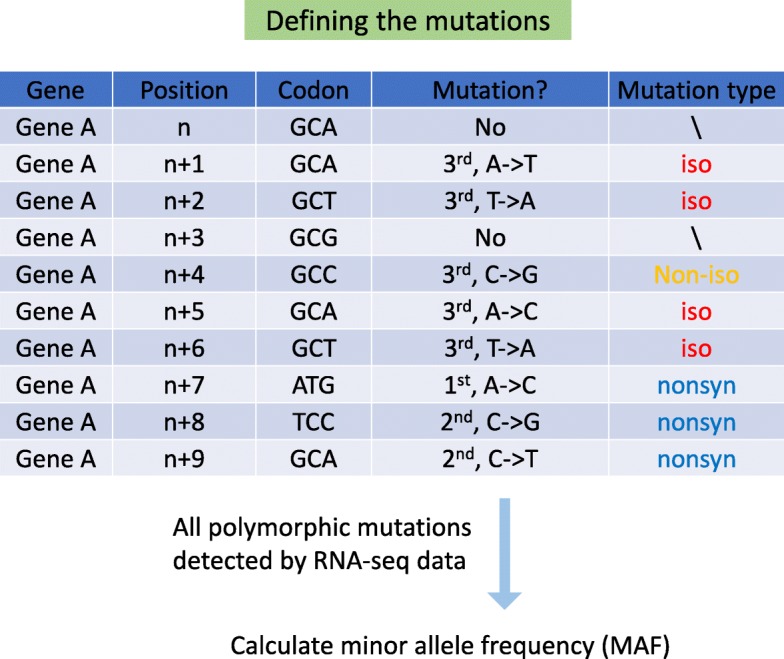
Pipeline of the process of defining types of mutations. Each mutation in the CDS must belong to one of three categories: isoaccepting, nonisoaccepting or nonsynonymous (if nonsense mutations are not considered). The relationship is determined by the codon after the mutation versus the codon before the mutation. For each mutation, the minor allele frequency (MAF) is then calculated

If a codon has a mutation, the results could only be (1) isoaccepting, (2) nonisoaccepting, (3) nonsynonymous or (4) nonsense. The isoaccepting and nonisoaccepting mutations belong to the synonymous category. Thus, we classified all of the detected polymorphic mutations according to the relationship between the codon after mutation versus the codon before mutation (Fig. 2 and Methods). Among the 24,323 polymorphic mutations, 9423 are synonymous (6964 are isoaccepting and 2459 are nonisoaccepting), 14,511 are nonsynonymous and 389 are nonsense mutations (Additional file 1: Figure S1). We next calculated the minor allele frequency (MAF) detected by the RNA-seq data (Methods). In brief, if a mutation has a level x > 0.5, then the MAF should be 1-x. Only bi-allelic positions were considered.

### Purifying selection on the nonsynonymous and nonsense mutations

Based on the number of different types of mutations we mentioned above (Additional file 1: Figure S1), we found that the MAF spectrum detected by the RNA-seq data exhibits a pattern of nonsense < nonsynonymous < synonymous (Table 2), which indicates the suppression of nonsense and nonsynonymous mutations. In fact, these patterns are not novel because the theory is already well-established and it is conceivable that the majority of nonsynonymous or nonsense mutations are non-adaptive. However, it is important for us to show this result to prove that our data and methodology are reliable and valid.

**Table 2:** Median minor allele frequency (MAF) of mutations

Mutation type	Nonsynonymous	Nonsense	Synonymous
Median frequency	0.212	0.128	0.249

### Parsing the mutations according to the codon context

Given the polymorphic synonymous mutations we detected, our next step was to classify these mutations according to the codon context. Taking the maize genome for instance, we obtained 39,254 unique coding genes. These 39,254 unique CDSs in total contain 13,958,446 codons. If we look at the relationship between a focal codon and the upstream codon (i.e., the environment/context) among these 13,958,446 codons, 334,757 are isoaccepting, 903,994 are nonisoaccepting and 12,680,441 are nonsynonymous (Additional file 1: Figure S1). We intended to divide all polymorphic synonymous sites (9423 mutations) according to contextual information. However, if the context of a focal codon had a polymorphism (which is relatively few), the codon itself was not considered.

### Selection on isoaccepting or nonisoaccepting mutations is region dependent: isoaccepting mutations are favored in isoaccepting stretches

Synonymous mutations were further classified into isoaccepting and nonisoaccepting mutations. We would hypothesize that the deleterious effects of nonsynonymous or nonsense mutations are “context independent”, because wherever they take place, they would cause AA changes (nonsynonymous) or introduce a premature stop codon (nonsense mutation). In contrast, as mentioned in the Background, isoaccepting stretches (codon co-occurrence) are advantageous due to the rapid recharging and reuse of tRNAs, so that isoaccepting mutations are only advantageous when they take place in these codon co-occurring regions to maintain the relationship between neighboring codons. In other words, whether an isoaccepting or nonisoaccepting mutation is advantageous or not is “region dependent” or “context dependent”.

**Fig. 3:**
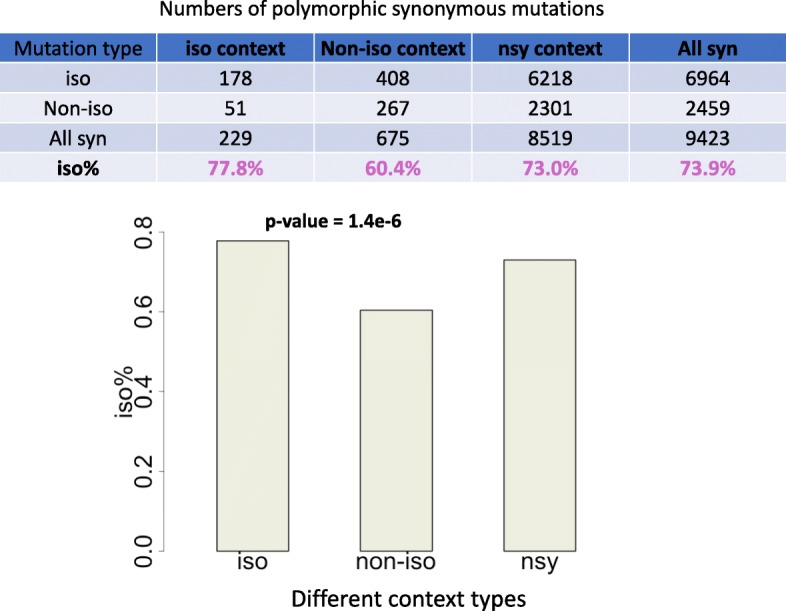
Isoaccepting mutations are favored in an isoaccepting context. The numbers of different types of mutations in each category are shown. The fractions of isoaccepting to all synonymous mutations to all synonymous mutations (iso%) in each context are plotted. The *p*-value was calculated by Fisher’s exact test. The *p*-value was compared between the iso and noniso context

We have already classified polymorphic synonymous sites into three categories according to context. We calculated the fraction of isoaccepting mutations to all synonymous mutations (iso%) in each region (Fig. 3). We could see that the iso% in the isoaccepting context was significantly higher than the iso% in a nonisoaccepting context, and the iso% in nonsynonymous context was intermediate (Fig. 3).

These observations could be explained as follows: isoaccepting mutations are favored in an isoaccepting context, nonisoaccepting mutations are favored in a nonisoaccepting context, while in other regions such as a nonsynonymous context, all synonymous mutations (iso- or nonisoaccepting) are equally favored.

### Frequency spectrum further supports the advantage of isoaccepting mutations in an isoaccepting context

We parsed the MAF spectrum of the polymorphic mutations from the RNA-seq data (Methods). We mentioned that the MAF detected by RNA-seq data exhibited a pattern of nonsense < nonsynonymous < synonymous mutations (Table 2), suggesting that our data and methodology can reliably detect the selection patterns.

**Fig. 4:**
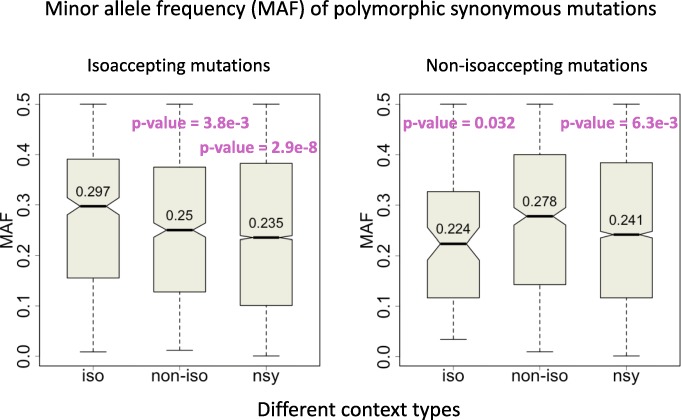
Signals of natural selection inferred from the MAF spectrum of polymorphic mutations. Mutations are classified according to the codon context. The MAF distributions of different types of mutations are plotted. The p-value was calculated by Wilcoxon rank sum test. *P*-values in the left panel are compared with the iso context. P-values in the right panel are compared with the noniso context

For polymorphic synonymous sites in different codon contexts, we profiled the MAF of isoaccepting or nonisoaccepting mutations in these regions (Fig. 4). Interestingly, the MAF spectrum of isoaccepting mutations was significantly higher in an isoaccepting context than other contexts (Fig. 4). Similarly, the MAF of nonisoaccepting mutations was significantly higher in a nonisoaccepting context than other contexts (Fig. 4). This is strong evidence supporting that isoaccepting mutations in an isoaccepting context are positively selected. The same theory goes for nonisoaccepting mutations in a nonisoaccepting context.

### The pattern is robust when CpG regions are excluded

There is a potential bias (or confounding factor) such that the mutation spectrum might be different in the CpG regions. Therefore, we need to ensure that our observation is not caused by the property of these CpG regions. We calculated the base content of A, C, G, T and CpG in these genomes. CpG is defined as a CG di-nucleotide in the genome. We found that the observed content of CpG is higher than the expected CpG frequency (Additional file 1: Figure S3). We discarded the mutations in the CG di-nucleotide (Additional file 1: Figure S3). The pattern is robust if we only use the mutations in non-CpG regions: (1) isoaccepting mutations are favored in an isoaccepting context and nonisoaccepting mutations are favored in a nonisoaccepting context (Additional file 1: Figure S3) and (2) the frequency spectrum further supports the advantage of isoaccepting mutations in an isoaccepting context (Additional file 1: Figure S3).

## Discussion

At a genome-wide level, we have systematically characterized the codon co-occurrence bias in maize. Intriguingly, in the fraction comparison (Fig. 3), we observed that the fraction of isoaccepting mutations (iso%) is higher in an isoaccepting context than a nonisoaccepting context. However, to perform a formal test on positive/negative selection, we must perform a comparison on the frequency spectrum.

The limitation of this study is that we used RNA-seq rather than DNA-seq data to perform variant calling. On one hand, the frequency spectrum (Table 2) demonstrated that the variant sites are reliable. On the other hand, we conducted linkage disequilibrium (LD) analysis to prove that the variation sites are likely to be DNA mutation sites rather than RNA level alterations or sequencing errors. According to the protocol for calculating the LD between two mutation sites [23] (Additional file 1: Figure S4), we calculated the pairwise R^2^ values between any pair of mutation sites that are detectable by a single RNA-seq read (Methods). We found that the majority of pairwise R^2^ values calculated from the RNA-seq data are strong (median value = 1, Additional file 1: Figure S4). Since a greater R^2^ indicates stronger linkage and only DNA mutations rather than sequencing errors could have such a strong linkage, our result simply suggests that the variant sites called from RNA-seq data are likely to be authentic DNA mutations.

In this study, we revealed that the codon co-occurrence bias in the genome is selectively favored and maintained by natural selection. The advantage of this co-occurrence bias might be the rapid recharging and reuse of tRNAs to increase the efficiency of translation elongation. The elevated fitness caused by the faster recharging of tRNA leads to more efficient decoding and a faster translation elongation rate. Although the key factor determining protein abundance is translation initiation rather than the elongation process, the flexible fine-tuning of translation elongation speed could also be advantageous and subject to natural selection. Intriguingly, it has been established that the biological significance of codon usage bias (CUB) is modulating the local translation elongation rate [6–9]. If we take codon co-occurrence bias into consideration, the purposes of these different types of codon biases all result in fine-tuning the translation elongation process. It is possible that the switches between different synonymous codons were originally designed to slightly modulate the translation process.

Another potential limitation in our work is that a mutation may affect not only the rate of translation elongation but also the rate of transcription elongation, splicing efficiency, localized and long distance intramolecular base-pairing within an mRNA, and DNA-histone interactions and thus genome structure. Although at this stage it is difficult to sort out the degree by which any one mutation would have on these “other aspects”, it is important to point out that our observed consequences are likely a mixture of the combinatory effects of multiple factors. Hopefully, if there are quantitative measurements on the effect of mutations on those “other aspects”, then a multiple regression analysis could be performed to parse the relative contribution of multiple factors to the observed patterns. For example, by fitting a simplified linear model of *Y* ~ *X*_*1*_ + *X*_*2*_ + ... + *X*_*n*_ where *Y* = the observed biases in the mutation pattern and *X*_*1*_, *X*_*2*_, ... *X*_*n*_ represents the factors such as translation elongation rate, transcription elongation rate, splicing efficiency, intramolecular base-pairing of mRNA, and DNA-histone interactions, one could quantitatively determine the regression efficiency, and thus the relative contribution of multiple factors.

Moreover, although isoaccepting codons can accept the same tRNA, the kinetics of codon-anticodon interactions may influence which nonisoaccepting mutations are actually observed. This could be the next level of analysis. Although this could be an issue related to maintaining efficient translation elongation as an aspect of the rapid recharging notion, it also would be subject to the same potential effects that it may have on transcription, splicing, intramolecular mRNA base-pairing, and genome structure, as discussed above. It is also worth noting that the rate of translation elongation is not always optimal for all genes and that is why terminologies such as CAI (codon adaptation index) or tAI (tAI adaptation index) were created to measure this aspect [24, 25]. If rapid recharging does actually occur in vivo and thus facilitates the efficient translation for many genes, this may not be the case, nor even desirable, for all genes as the rate of translation elongation may be one means, and in some cases is such a means, to regulate the rate of protein expression. That the expression of all genes may not be optimized for efficient translation elongation may contribute to the extent to which nonisoaccepting mutations are observed.

It remains unsolved whether the iso−/nonisoaccepting mutations in an iso−/noniso context are at equilibrium, or whether one side is disrupted more frequently than the other side. If there are genome data for a population collected from different generations, one could investigate whether the fraction of iso−/nonisoaccepting mutations in an iso−/nonisoaccepting context keep changing along generations or if they are stabilized after a particular time point. This would help to better understand the dynamics of the mutations in different genomic regions.

In summary, our work deepened the understanding of codon co-occurrence bias in plants from the perspective of evolutionary biology and might provide novel perspectives to help solve the riddles related to angiosperm evolution.

## Conclusions

By investigating the polymorphic and fixed mutations in four plant genomes, we revealed that in the regions with co-occurring isoaccepting codons, natural selection has attempted to maintain this co-occurrence pattern by suppressing the nonisoaccepting mutations in these regions. However, if the consecutive synonymous codons are nonisoaccepting, the mutations in these regions tend to be nonisoaccepting and therefore attempt to switch these nonisoaccepting codons to become isoaccepting. Our results support the model of rapid recharging and reuse of tRNA. This uneven distribution of isoaccepting mutations seems to be a relic shaped by natural selection.

## Methods

### Data collection

The path for the CDS sequences of maize is (also see Additional file 1: Figure S1): ftp://ftp.ensemblgenomes.org/pub/release-43/plants/fasta/zea_mays/cds/. The tRNA data of *Zea mays* were downloaded from the Genomic tRNA database (GtRNAdb). The SRR IDs of the RNA-seq data are: *Zea mays* (five libraries, SRR8560815-SRR8560819). All SRR samples were sequenced from the roots of the plants.

### Processing the RNA-seq data to infer the polymorphic sites in CDS

Since we do not care about the mutations in noncoding regions, the reference CDS sequences and deep-sequenced RNA-seq data are sufficient to call the polymorphic variants in coding regions. We mapped the RNA-seq reads to the *Zea mays* CDS sequences using Bowtie2 (version 2.2) [26]. Considering the multiple mapped reads caused by different isoforms of the same gene, only the longest CDS of each gene was chosen. We called variants using SAMtools (version 1.5) “mpileup” with default parameters [27]. To avoid the false positive variants caused by sequencing errors (low frequency in RNA-seq), only the variant sites with levels between 0.02 and 0.98 were regarded as candidate polymorphic mutations. If multiple mutations took place within the same codon or consecutive codons, these cases were not included in the following analyses. The reason for this filtering step is that in the following analyses we classified the mutations according to their codon context. We next defined the minor allele frequency (MAF) of each site. If a mutation has a level x > 0.5, then the MAF = 1-x. Only bi-allelic sites were included. In the RNA-seq data we analyzed, the median levels of different types of mutations were approximately 0.2~0.3 (based on the reference genome); even when we calculated the MAF, the median value only slightly decreased.

### Definition of isoaccepting codons

A codon can pair with one to multiple (up to three) different anticodons and an anticodon can pair with one to multiple (up to three) different codons [24]. For a given codon, its isoaccepting codon(s) is (are) the synonymous codon(s) that could pair with the same anticodon (tRNA) [2]. The list of all possible isoaccepting codon(s) for each of the 59 sense codons (excluding ATG, TGG and three stop codons) is given in Table 1. In different organisms, the groups of isoaccepting codons might be slightly different due to some tRNAs (anticodons) being absent in the species. An example of isoaccepting relationships among alanine codons is shown in Additional file 1: Figure S2.

### Definition of isoaccepting and nonisoaccepting mutations

Isoaccepting mutations are those mutations that do not change the decoding anticodon (tRNA) of a codon (Fig. 1a). Nonisoaccepting mutations are those mutations that change the decoding anticodon (tRNA) of a codon. In this study, nonisoaccepting mutations only refer to those synonymous mutations that change the decoding tRNA.

### Definition of codon context

The relationship between the two consecutive codons could be (1) isoaccepting, (2) nonisoaccepting but synonymous, or (3) nonsynonymous. The pipeline for defining iso/nonisoaccepting/nonsynonymous context is as follows: We extracted all polymorphic synonymous mutations in CDS. Polymorphic neighboring sites were excluded. The relationship between the focal codon and the previous codon determined the context type (environment type).

### Calculating the linkage disequilibrium (LD) from sequencing data

Presume that loci 1 and 2 are two mutation sites. In the RNA-seq data, there are a total of N reads that could cover both sites (Additional file 1: Figure S4), and N = RR + RM + MR + MM, where R stands for the reference allele and M stands for the mutation. For example, RM is the number of reads that detects the reference allele on site 1 and detects the alternative allele on site 2. Next, let P1 = (RR + RM)/N, Q1 = (MR + MM)/N, P2 = (RR + MR)/N, Q2 = (RM + MM)/N, such that the LD parameter D is calculated as D = (RR*MM - RM*MR)/N^2^. Finally, the R-squared value is calculated as R^2^ = D^2^/(P1*Q1*P2*Q2). A greater R^2^ indicates a stronger linkage. The R^2^ should range from 0 to 1. In Additional file 1: Figure S4, we present an example of a pairwise linkage plot for 13 mutation sites in gene Zm00001d031523_T001. The positions of these mutation sites are shown in the plot. The global distribution of the pairwise R^2^ is displayed as a boxplot (Additional file 1: Figure S4).

### Statistical analysis and code availability

All statistical analyses (correlation tests, Fisher’s exact tests) and the graphic work were conducted in R environment (http://www.R-project.org/). The codes used in this study are available by request.

## Supplementary information

**Additional file 1: Figure S1.** Locations of the CDS sequence files of the plant species used in this study. The basic statistics such as the number of genes and codons in maize, and the numbers of detected different mutation types in maize are also displayed. **Figure S2.** An example of how to define the isoaccepting codon(s) of a given codon. The four Ala codons are taken as an example. **Figure S3.** The observed patterns are robust when excluding the CpG regions as a confounding factor. Only mutations outside the CpG regions were considered in this case. Significance was defined by Fisher’s exact test and the Wilcoxon rank sum test. **Figure S4.** Linkage disequilibrium (LD) analysis of the pairwise mutation sites called from the RNA-seq data. An example of a (pairwise) linkage plot is shown for 13 mutation sites in gene Zm00001d031523_T001. The positions of these mutation sites are given in the plot.

## Data Availability

The datasets supporting the conclusions of this article are available from the NCBI or Ensembl website (as described in the Methods section).

## Abbreviations

CAI: Codon adaptation index
CDS: Coding sequence
CUB: Codon usage bias
mRNA: messenger RNA
tAI: tRNA adaptation index
tRNA: transfer RNA
