# Supplementary material for: Parsing the synonymous mutations in the maize genome: isoaccepting mutations are more advantageous in regions with codon co-occurrence bias
Source: BMC Plant Biol. 2019 Oct 14;19:422. doi: 10.1186/s12870-019-2050-1 (PMC6791113; doi:10.1186/s12870-019-2050-1)
Supplement: Supplementary file 1 — Additional file 1: Figure S1. Locations of the CDS sequence files of the plant species used in this study. The basic statistics such as the number of genes and codons in maize, and the numbers of detected different mutation types in maize are also displayed. Figure S2. An example of how to define the isoaccepting codon(s) of a given codon. The four Ala codons are taken as an example. Figure S3. The observed patterns are robust when excluding the CpG regions as a confounding factor. Only mutations outside the CpG regions were considered in this case. Significance was defined by Fisher’s exact test and the Wilcoxon rank sum test. Figure S4. Linkage disequilibrium (LD) analysis of the pairwise mutation sites called from the RNA-seq data. An example of a (pairwise) linkage plot is shown for 13 mutation sites in gene Zm00001d031523_T001. The positions of these mutation sites are given in the plot. [file 12870_2019_2050_MOESM1_ESM.pdf]

Species	CDS sequence	RNA-seq data
Zea mays	ftp://ftp.ensemblgenomes.org/pub/release-43/plants/fasta/zea_mays/cds/	SRR8560815-SRR8560819

Number of mutations detected in the whole CDS regions

Type	Total	Isoaccepting	Non-isoaccepting	Non-synonymous	Nonsense
Polymorphic	24323	6964	2459	14511	389

Species	Number of unique genes	Number of unique codons
Zea mays	39254	13958446

Relationship between the focal codon and the front codon

Start codon	Isoaccepting	Non-isoaccepting	Nonsynonymous
39254	334757	903994	12680441

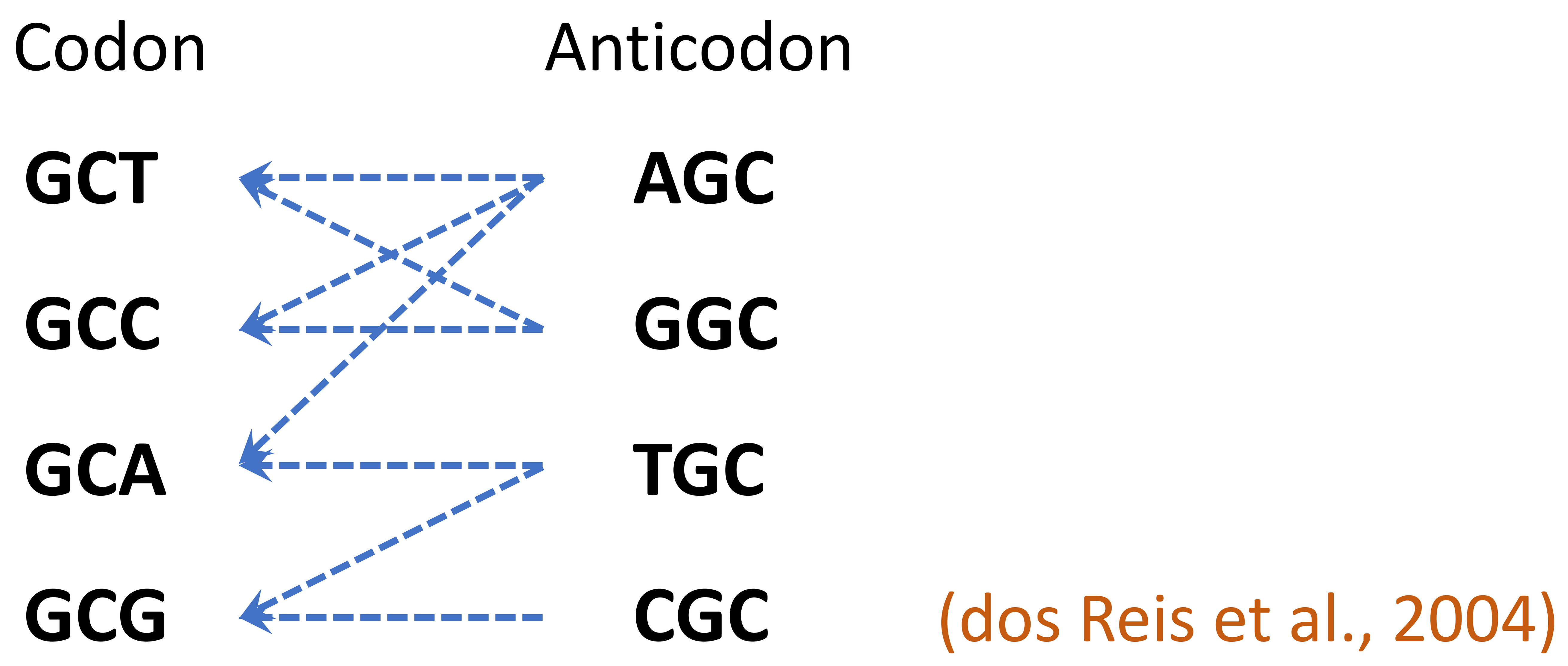

Ala Codon	Isoaccepting codon(s)
GCA	GCT/GCC/GCG
GCC	GCT/GCA
GCG	GCA
GCT	GCA/GCC

Fraction of nucleotide in CDS

Species	A (%)	C (%)	G (%)	T (%)	CpG (%)	C(%)*G(%)
Zea mays	23.3	26.7	28.4	21.6	13.6	7.6

Non-CpG region in CDS

Minor allele frequency (MAF) of polymorphic synonymous mutations

Item	**iso context**	**Non-iso context**	**nsy context**	**All syn**
iso%	77.5%	61.1%	73.2%	73.6%
MAF iso	0.292	0.253	0.240	0.243
MAF non-iso	0.229	0.275	0.245	0.246

Different context types

RNA-seq reads

R: reference allele.

M: mutation.

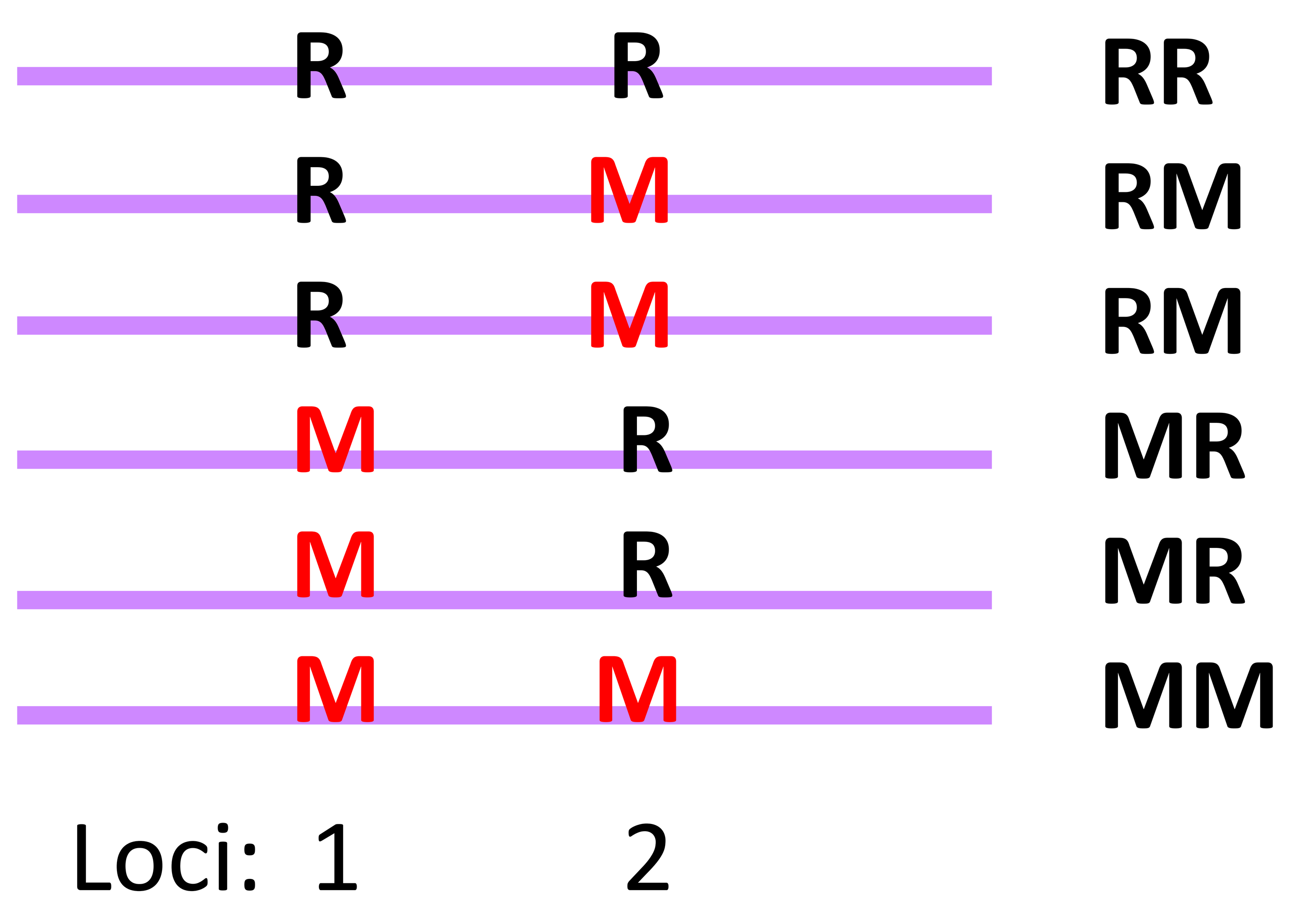

$$N = RR + RM + MR + MM$$
$$P1 = (RR + RM)/N$$
$$Q1 = (MR + MM)/N$$
$$P2 = (RR + MR)/N$$
$$Q2 = (RM + MM)/N$$
$$D = (RR*MM - RM*MR)/N^2$$
$$R^2 = D^2/(P1*Q1*P2*Q2)$$

Zm00001d031523_T001

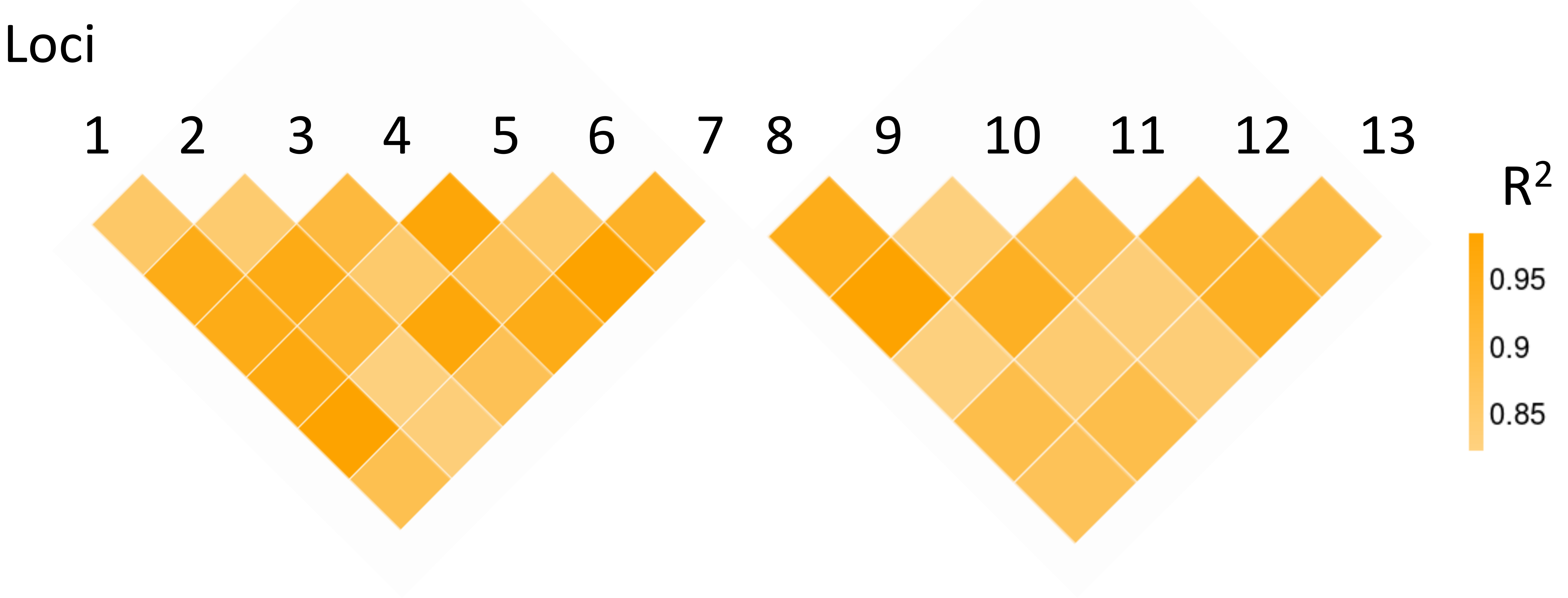

All detected polymorphic mutations

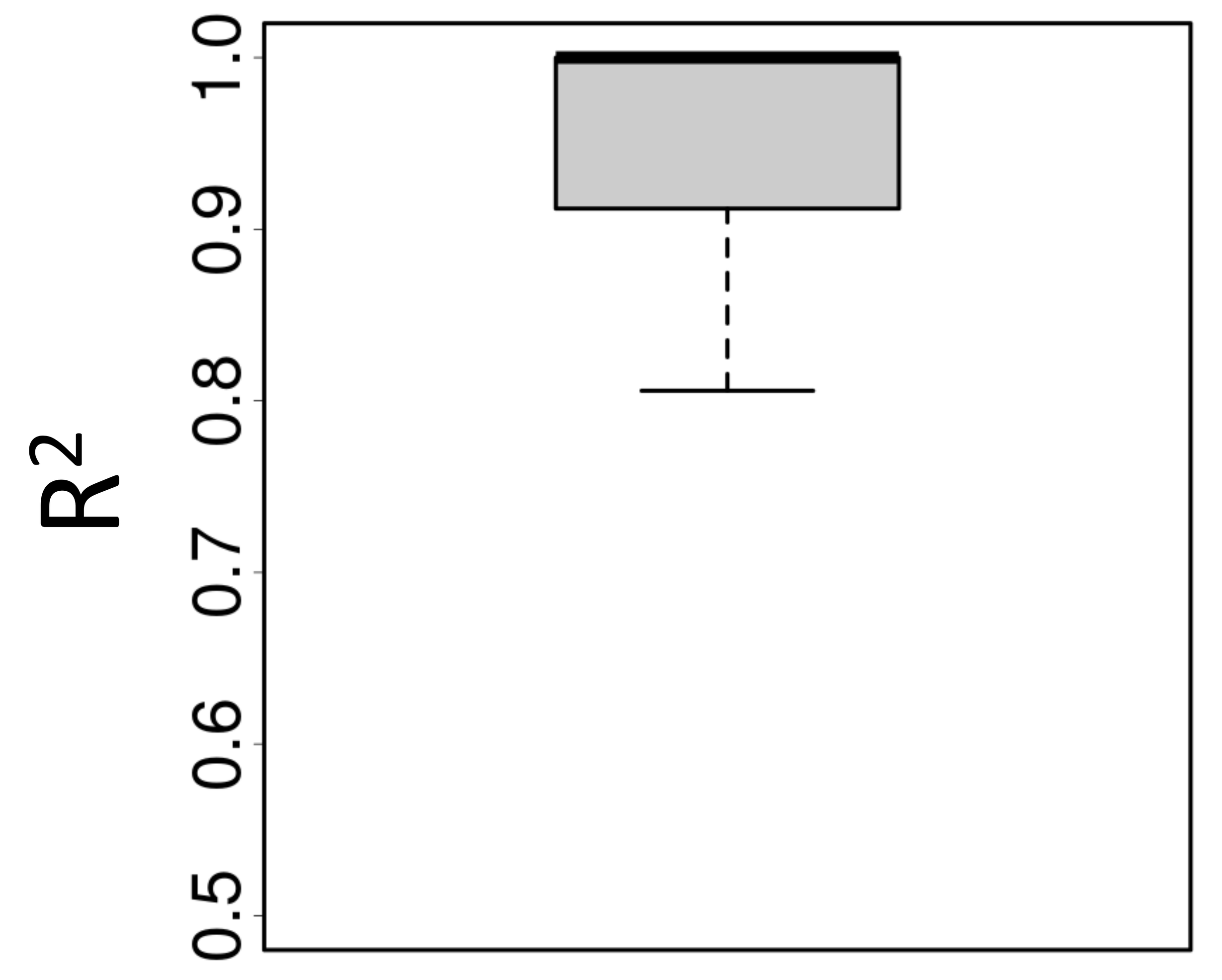

Loci 1-13: CDS position 240, 248, 254, 255, 256, 271, 287, 291, 299, 308, 315, 319, 326
